# SEU-Hardened High-Speed SRAM Design with Self-Refresh and Adjacent-Bit Error Correction

**DOI:** 10.3390/mi17030342

**Published:** 2026-03-11

**Authors:** Tianwen Li, Jianbing Tian, Jingli Qi

**Affiliations:** 1 Beijing Aerospace Shenzhou Intelligent Equipment Technology Co., Ltd., Beijing 100086, China; 2 Beijing Sunwise Space Technology Ltd., Beijing 100190, China

**Keywords:** single-event upset (SEU), SRAM, error correction code (ECC), self-refresh mechanism, adjacent bit errors, dynamic circuit

## Abstract

This paper proposes a high-speed static random access memory (SRAM) architecture that integrates a self-refresh mechanism with a novel single error and adjacent-bit errors correction (SEABEC) scheme to enhance resilience against single-event upsets (SEUs) in radiation-prone environments. By leveraging extended Hamming coding and dynamic circuits, the design achieves a 29.1% RW speed improvement, reduces SEU cross-section by one order of magnitude, and incurs a 29.8% area overhead and a 95.2% dynamic power increase of the ECC module, leading to an overall chip area increase of ~14.2% compared to static logic-based RH SEC-DED SRAM. Radiation experiments validate superior tolerance across a LET range of 1.63–21.8 MeV·cm^2^/mg, demonstrating nearly doubled SEU resilience compared to conventional SEC-DED-based designs. This work balances error correction capabilities with system efficiency, making it suitable for high-reliability applications in space electronics and advanced processors.

## 1. Introduction

Advancements in microelectronics have led to substantial improvements in on-chip data processing performance and storage capacity. Memory is now extensively integrated into a wide range of very large-scale integrated circuits [1,2,3], rather than being limited to independent memory components. Within general-purpose processors, including Field Programmable Gate Array (FPGA), Central Processing Unit (CPU), and Digital Signal Processor (DSP), as well as System-on-Chip (SoC) platforms, memory accounts for a significant share of the total chip area, typically ranging from approximately 40 to 70%, and therefore represents a core system component [4,5]. Memory technologies encompass multiple categories, each suited to specific application scenarios. Among these options, static random access memory (SRAM) provides advantages in operating speed, power efficiency, service lifetime, and stability, which render it more appropriate than alternative memory types for high-performance general-purpose processors and SoC [6,7].

Currently, the majority of electronic systems incorporate memory components to store user data, and the reliability of these memories directly impacts the normal operation and functionality of the overall system [8]. Relative to other circuit modules, memory structures exhibit greater vulnerability to faults, encompassing both soft errors and hard errors. Existing studies report that the occurrence rate of soft memory mistakes exceeds that of hard errors by more than 5000 times, indicating that soft-error phenomena dominate reliability issues in large-capacity memory. In electronic systems, soft errors arise predominantly within memory units [9].

As a fundamental element for instruction and data storage, memory is extensively deployed in electronic systems, including satellites, spacecraft, and deep-space probes. In space environments, memory devices are exposed to cosmic radiation and high-energy particles, which can induce single-event upsets (SEUs) during operation. When such events corrupt stored data, the resulting errors may trigger malfunctions in spaceborne electronic equipment or cause complete system failures, ultimately jeopardizing mission success [10]. For this reason, improving memory resilience against SEUs is essential for ensuring the survivability of space electronic systems [11,12].

Existing approaches for mitigating SEUs in memory primarily fall into two categories: information redundancy and hardware redundancy. Information redundancy introduces additional error correction codes (ECCs) into user data, including Hamming codes, parity schemes, and cyclic codes [13]. An increase in the number of redundant information bits generally strengthens error detection and correction (EDAC) capability. Hardware redundancy, in contrast, replicates memory resources to construct dual- or triple-redundant storage architectures. Owing to the substantial area overhead associated with hardware-based redundancy, ECC techniques have been widely adopted in high-reliability electronic system design, as they offer a more favorable balance between fault tolerance and silicon cost [14,15].

As CMOS device feature sizes enter the nanoscale era, the number of devices per unit area on chips continues to increase. The rapid escalation in device density results in reduced distances between memory cells, making it increasingly likely for a single high-energy particle strike to induce multiple bit flips in adjacent storage units. Single-particle irradiation experiments demonstrate that at a 90 nm process node, the probability of two-bit single-event multiple-bit upsets (SEMBUs) occurring simultaneously reaches approximately 60%. Consequently, the probability and severity of SEMBU events escalate with shrinking feature sizes, posing a critical reliability challenge for deep-submicron memory systems. While Hamming codes capable of single-error correction and double-error detection (SEC-DED) have been widely adopted in SEU-hardened memory designs, these codes are limited to correcting one erroneous bit and detecting two errors within a byte, lacking the capability to recover from double-bit data errors. Although SEC-DED codes offer minimal area overhead, their inadequacy becomes apparent as adjacent bit flip probabilities rise, necessitating the development of advanced error correction algorithms to enhance memory robustness against adjacent-bit errors in advanced processes.

A novel ECC scheme is introduced to strengthen correction capability without increasing the number of parity bits, thereby further enhancing the reliability of large-capacity memory during data storage and read/write (RW) operations. The proposed algorithm improves tolerance to single-bit error and adjacent-bit errors induced by SEU effects. In addition, the paper surveys recent advances in memory reliability research and outlines the principal contributions of this work, including the combined use of self-refresh techniques and single-error and adjacent-bit errors correction (SEABEC).

## 2. Chip Architecture and Circuit Design

**Figure 1:**
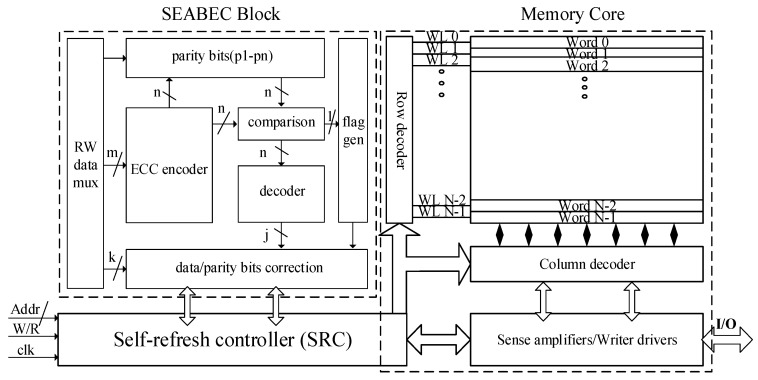
Architecture diagram of SEU-hardened memory.

The overall block diagram of the SEU-hardened SRAM is shown in Figure 1,which consists of memory core and SEABEC block, integrating multiple key circuits to achieve high efficiency, robust protection, and flexible operation. The proposed design adopts a 130 nm CMOS fabrication process and employs Cadence Virtuoso for circuit simulation and layout development. The implementation uses conventional six-transistor SRAM cells, dynamic logic, etc. The methodological workflow involves three stages: development of the SRAM storage array and associated peripheral circuitry, implementation of the ECC unit, and integration of self-refresh mechanisms. The following sections provide detailed implementations of each major component.

## 3. Circuit Design

### 3.1. Memory Core

The memory core, designed based on mature and well-established technologies for SRAM modules, is composed mainly of the storage array, address decoders, a precharge unit, an output sense amplifier, and RW control logic. The architecture supports random access to individual memory cells, which underlies its classification as random access memory. Additionally, uniform access latency is maintained for all cells, ensuring that data access speed is independent of the physical position of a given cell within the array.

### 3.2. SEABEC Block

As shown in Figure 1, the SEABEC block is primarily composed of error correction coding, a decoding module, an error correction module, and a self-refresh module.

(1) The novel ECC algorithm

To address adjacent-bit error correction, conventional approaches require bit interleaving during layout design, which significantly complicates global routing, enlarges chip area, and degrades performance. For instance, a standard 16-bit word without interleaving requires a wiring width of 16 tracks, whereas interleaving escalates this to 32 tracks. Given the reduced device pitches in deep-submicron technologies that amplify the likelihood of multi-bit upsets within a single byte, this work proposes a novel 16-bit ECC encoding scheme capable of correcting arbitrary single-bit errors and adjacent-double-bit errors.

**Figure 2:**
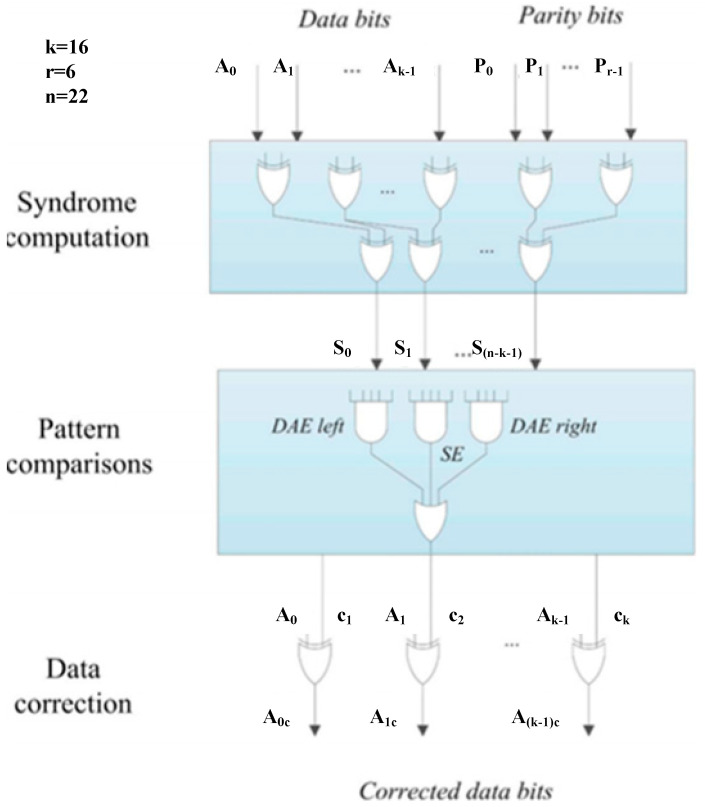
Block diagram of a SEC-DAEC decoder [3].

The proposed SEABEC code is constructed with a parity check matrix (H-matrix) that satisfies two core design criteria: (1) all columns are distinct and non-zero; (2) the XOR result of any two adjacent columns is unique and differs from all individual columns of the matrix. This structural design ensures that single-bit errors and double-adjacent-bit errors generate distinct syndrome values, enabling their accurate identification and correction. For an n-bit code word, the SEABEC code is capable of correcting n single-bit error patterns and (n − 1) double adjacent-bit error patterns. As illustrated in Figure 2, the typical SEABEC decoder consists of three functional blocks: (1) Syndrome calculation: recalculates the parity check bits from the read data and performs an XOR operation with the pre-stored parity bits to generate the syndrome vector. (2) Syndrome comparison: for each data bit, verifies whether the generated syndrome matches the predefined single-error (SE) pattern or either of the two double-adjacent error (DAE) patterns (left-adjacent and right-adjacent) corresponding to that bit, and generates a correction signal if a match is detected. (3) Error correction: implements error correction by XORing the erroneous data bit with the corresponding correction signal to recover the error-free data.

The parity check H-matrix for the novel ECC scheme is defined as

$$H=\left[\begin{array}{cccccccccccccccccccccc} 0 & 1 & 1 & 1 & 0 & 1 & 1 & 1 & 0 & 0 & 1 & 0 & 1 & 1 & 0 & 1 & 1 & 0 & 0 & 0 & 0 & 0\\ 1 & 1 & 0 & 0 & 0 & 1 & 1 & 1 & 1 & 0 & 0 & 1 & 0 & 1 & 1 & 0 & 0 & 1 & 0 & 0 & 0 & 0\\ 0 & 0 & 1 & 0 & 1 & 1 & 0 & 0 & 0 & 1 & 0 & 1 & 1 & 1 & 0 & 1 & 0 & 0 & 0 & 0 & 1 & 0\\ 1 & 0 & 0 & 1 & 0 & 1 & 0 & 1 & 0 & 1 & 0 & 0 & 1 & 0 & 1 & 0 & 0 & 0 & 0 & 1 & 0 & 0\\ 1 & 0 & 1 & 0 & 1 & 0 & 1 & 0 & 1 & 0 & 1 & 0 & 0 & 0 & 0 & 0 & 0 & 0 & 0 & 0 & 0 & 1\\ 0 & 1 & 1 & 1 & 1 & 1 & 0 & 0 & 0 & 1 & 0 & 0 & 1 & 1 & 1 & 1 & 0 & 0 & 1 & 0 & 0 & 0 \end{array}\right]$$

The corresponding system of encoding equations is

P0 = A1⊕A2⊕A3⊕A5⊕A6⊕A7⊕A10⊕A12⊕A13⊕A15(1)

P1 = A0⊕A1⊕A5⊕A6⊕A7⊕A8⊕A11⊕A13⊕A14(2)

P2 = A2⊕A4⊕A5⊕A9⊕A11⊕A12⊕A13⊕A15(3)

P3 = A0⊕A3⊕A5⊕A7⊕A9⊕A12⊕A14(4)

P4 = A0⊕A2⊕A4⊕A6⊕A8⊕A10(5)

P5 = A1⊕A2⊕A3⊕A4⊕A5⊕A9⊕A12⊕A13⊕A14⊕A15(6)

The syndrome, which is solely associated with the error pattern, can be used to determine the position of errors and correct them. During decoding, the check bits are recalculated from the data stored in the memory, and then an XOR operation is performed with the check bits stored in the memory to obtain the syndrome S. This process can be represented by the following equation:

S0 = A1⊕A2⊕A3⊕A5⊕A6⊕A7⊕A10⊕A12⊕A13⊕A15⊕P0(7)

S1 = A0⊕A1⊕A5⊕A6⊕A7⊕A8⊕A11⊕A13⊕A14⊕P1(8)

S2 = A2⊕A4⊕A5⊕A9⊕A11⊕A12⊕A13⊕A15⊕P2(9)

S3 = A0⊕A3⊕A5⊕A7⊕A9⊕A12⊕A14⊕P3(10)

S4 = A0⊕A2⊕A4⊕A6⊕A8⊕A10⊕P4(11)

S5 = A1⊕A2⊕A3⊕A4⊕A5⊕A9⊕A12⊕A13⊕A14⊕A15⊕P5(12)

The computed syndrome of the new algorithm demonstrates the relationship with single-bit error positions as outlined in Table 1, while its correspondence to adjacent-bit error patterns is illustrated in Table 2.

(2) Self-refresh module

The self-refresh module primarily receives external address, read/write control, and clock signals. It internally detects whether errors occur during each data read operation. Upon detecting an error flag, it rewrites the corrected data back to the storage array to prevent cumulative errors from rendering the ECC function ineffective.

(3) High-Speed Error-Correcting Circuit Design

**Figure 3:**
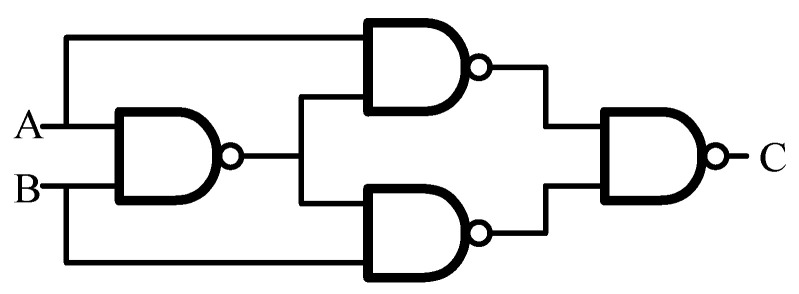
Gate-level implementation of XOR gate.

**Figure 4:**
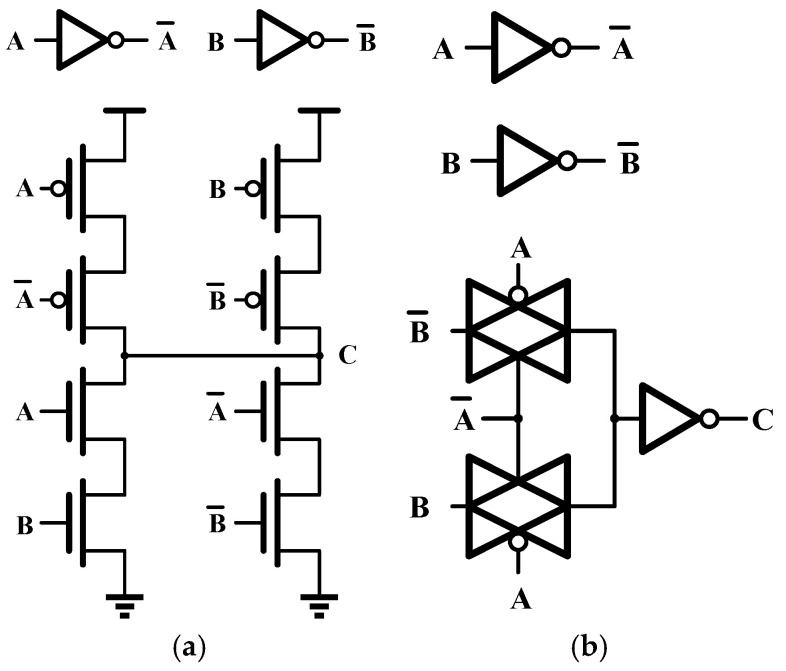

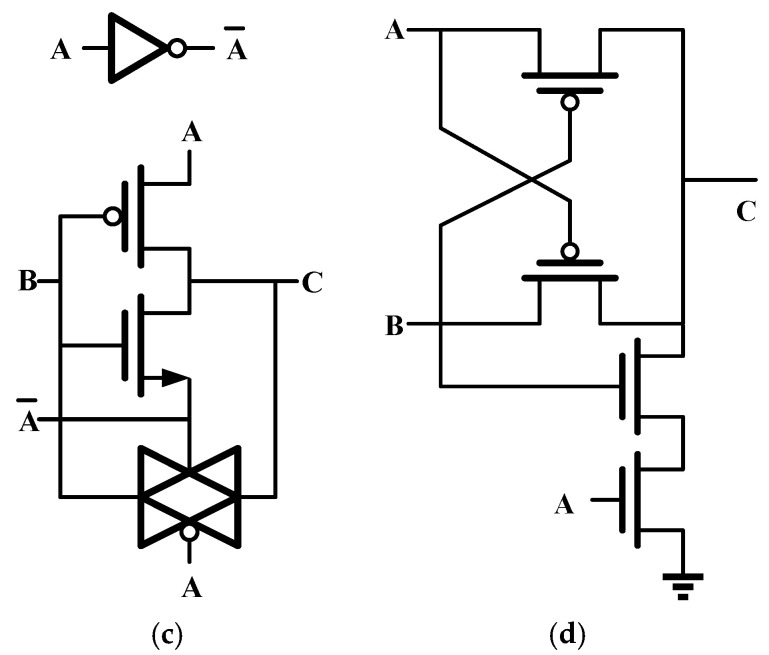
Different XOR gate circuit implementations: (**a**) Complementary logic implementation; (**b**) transmission gate implementation; (**c**) combination of transmission tube and transmission gate implementation; (**d**) transmission tube implementation.

The XOR gate constitutes the fundamental computational element in ECC circuitry. Conventional XOR gate designs based on static logic are generally unable to satisfy the requirements of high-speed and high-reliability memory systems. A gate-level realization of the XOR function is shown in Figure 3, while Figure 4 presents four representative XOR implementations. Figure 4a illustrates a CMOS logic realization, whereas Figure 4b depicts a transfer-gate-based structure that is simple and fast and is widely employed in standard cell libraries. Figure 4c shows an implementation that combines a transfer gate with a transfer transistor, commonly referred to as a reversible inverter configuration, which features a compact structure but suffers from incomplete logic level restoration. Figure 4d presents a four-transistor XOR implementation with high speed and low complexity; however, its output does not achieve full voltage swing.

**Figure 5:**
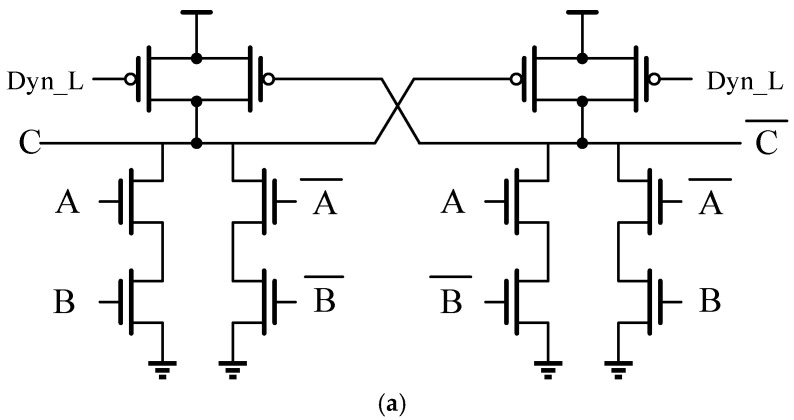

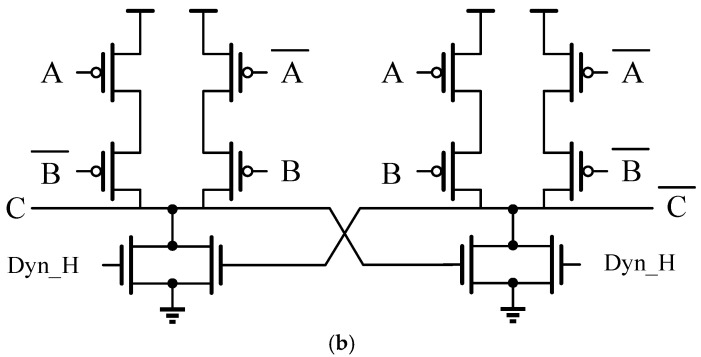
(**a**,**b**) Two types of dynamic XOR gates based on a differential structure. The notation *_bar represents the logical inverse of the corresponding signal * (i.e., A_bar = NOT A, B_bar = NOT B, C_bar = NOT C).

Conventional ECC circuits are typically realized through automated synthesis and layout flows, which offer simplicity and reduced development time. However, the standard cell libraries used in such tools rely on static CMOS logic and do not support dynamic circuit implementations. As a result, they fail to meet the high-speed requirements of ECC functions in high-performance memory designs. To address this limitation, a fully custom dynamic circuit approach is adopted for the ECC circuitry. Figure 5 illustrates the proposed differential dynamic XOR gate. To satisfy the monotonicity constraints inherent to dynamic logic, the custom design alternates between N-type differential dynamic XOR and P-type differential dynamic XOR structures, rather than appending inverters to a single dynamic stage to enforce monotonic behavior.

Compared with static CMOS XOR implementations, the dynamic logic structure exhibits reduced logical effort and smaller load capacitance, which translates into higher operating speed. Nevertheless, dynamic circuits rely on a precharge phase and therefore require completion of precharging before each XOR evaluation. If computation is initiated prior to the completion of precharge, incorrect results may occur. Consequently, accurate timing control of the precharge process is a critical requirement in dynamic circuit design.

**Figure 6:**
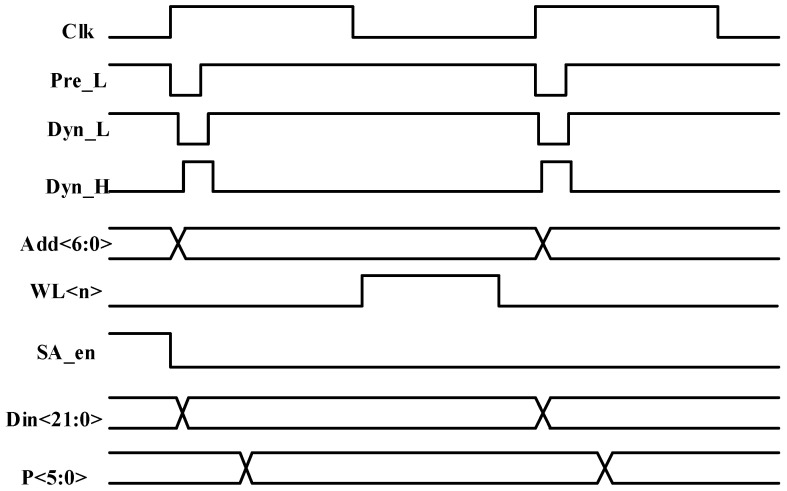
Write timing diagram.

**Figure 7:**
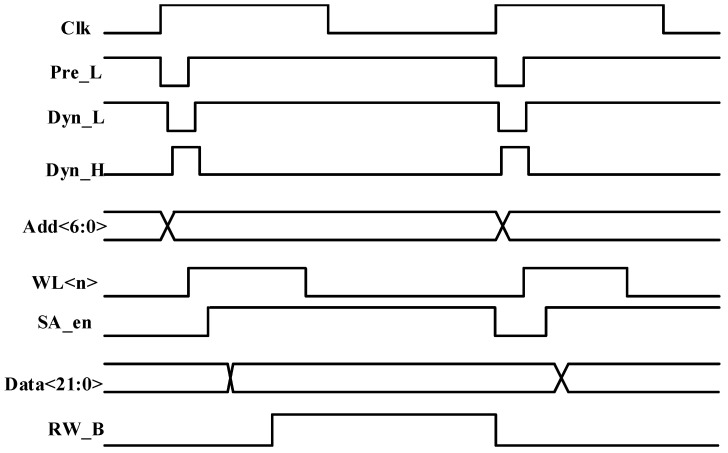
Read timing diagram.

The timing relationships among dynamic circuit precharge, read, and write operations are depicted in Figure 6 and Figure 7. When the rising edge of the clock arrives, the BL precharge signal Pre_L is asserted, initiating charging of the BLs to the supply voltage. During write operations, the EDAC module receives user input data. While the BL precharge signal is active, the input data of the encoding module is forcibly reset to zero. Subsequently, the dynamic circuit precharge signal is activated, initiating precharge of the entire error correction encoding module. In this phase, the N-type differential dynamic XOR gates are charged to the supply voltage, whereas the P-type differential dynamic XOR gates are discharged to ground. Because the dynamic gates employed in this design use footless logic, the precharge signal performs staged charging and discharging across the module. Specifically, charging or discharging of a subsequent stage begins only after completion of the preceding stage, which prevents race conditions inherent in footless logic. After the precharge of the EDAC module is completed, the forced reset of the encoding module input is released, and parity bit computation begins. Once parity generation is finished, the word line is enabled, and the user data, together with the computed parity bits, are written into the storage array, thereby completing the write operation.

During read operations, the data supplied to the EDAC module is obtained from the storage array. While the word line precharge signal is asserted, the input of the encoding module is forced to zero. The dynamic circuit charging and discharging sequence follows the same procedure as in write mode. After the EDAC module completes its precharge phase, the forced clearing of the encoding module input is released. Once word line precharging is completed, the word line is activated, and the selected storage cell begins discharging onto the BL. After a defined delay, the sense amplifier is enabled to sense the stored value, and the encoding module starts computing the parity bits based on the retrieved data. Upon completion of the parity computation, the error-type flag is evaluated to determine whether a SEU has occurred. When a single-bit error is detected, the refresh write control signal RW_B is asserted, initiating a write-back operation through the still-active word line to restore the corrected data at the original memory location. The corrected data is then delivered as output, thereby completing the read operation.

As shown in Table 3, the proposed dynamic SEABEC ECC circuit achieves a 29.1% read/write delay reduction with a 29.8% area increase and a 95.2% dynamic power rise, compared with the conventional static logic-based SEC-DED ECC circuit. The increased area and power, together with speed-optimized circuit design, result from SEABEC’s expanded functionality: it retains SEC-DED’s single-bit error correction and adds adjacent double-bit error correction for radiation hardening, which requires additional decoders and XOR gate arrays, and the dynamic circuit topology for high speed inherently introduces periodic precharge operations—these two factors jointly lead to the 95.2% dynamic power increment. We acknowledge that this power penalty is a critical consideration for spaceborne electronic devices with limited energy supply, yet it represents a reasonable engineering trade-off for high-performance, high-reliability on-board applications (e.g., on-board signal processors, deep-space probe data storage), where low-latency access and robust SEU resilience are prioritized over moderate-power overhead. Notably, the 95.2% power increase is limited to the ECC module; at the full chip level, the overall dynamic power rise is significantly diluted by the SRAM array and low-power peripherals, making the total power consumption compatible with typical spaceborne power budgets. For low-power priority space scenarios, the SEABEC circuit can be further optimized via low-voltage operation and dynamic power gating to reduce power overhead while retaining its core advantages. Though the SEABEC ECC circuit has higher area and power costs, its error correction capability is nearly doubled, significantly enhancing the SEU resilience of deep-submicron memory systems. Moreover, SEABEC is compatible with bit-interleaving techniques to further tolerate multiple consecutive bit errors—for example, two-byte bit interleaving enables four consecutive bit error correction with SEABEC. Designers must balance the trade-off between error correction capability and system performance across technology nodes, as enhanced correction typically brings larger area, higher power, or speed degradation. Thus, the design of radiation-hardened SRAM requires a pragmatic compromise between error correction performance, chip area, power consumption, and operating speed for radiation-prone applications, with flexible optimization strategies for different spaceborne application requirements.

### 3.3. Comparison of State-of-the-Art and Proposed Adjacent-Bit Errors Correction Codes

This section presents a comprehensive comparison between the proposed Single-Error and Adjacent-Bit Error-Correction (SEABEC) scheme and four state-of-the-art adjacent error-correction codes: Matrix Parity Code (MPC) [15], 3D Parity Check Code (3D) [16], Adjacent Error Detection and Correction Code (AEDAC) [17], and Single Error Correction–Double Adjacent Error Correction (SEC-DAEC) code [18]. As summarized in Table 4 for a 16-bit data width, the proposed SEABEC requires only 6 parity bits, resulting in a code word length of 22 bits and a code rate of 72.73%, which is significantly higher than MPC (51.6%), 3D (51.6%), AEDAC (53.33%), and SEC-DAEC (66.67%). While MPC, 3D, and AEDAC provide 2-bit error correction with 15, 15, and 14 parity bits, respectively, SEABEC achieves the same 2-bit correction capability with far fewer parity bits, leading to superior coding efficiency. In contrast, SEC-DAEC supports correction of partial 3-bit adjacent errors (78.02%) [18], but this extended capability comes at the cost of 8 parity bits, which is 33% more than the 6 bits required by SEABEC. The reduced parity overhead of SEABEC directly translates to simpler encoding/decoding logic, a smaller silicon area, lower power consumption, and higher speed potential, making it highly compatible with high-speed SRAM designs. In summary, the proposed SEABEC scheme strikes an optimal balance between reliability and efficiency, delivering robust 2-bit error correction with minimal resource overhead, and outperforms existing state-of-the-art schemes in terms of resource efficiency, implementation practicality, and cost–benefit ratio for most radiation-hardened memory applications.

### 3.4. Chip Layout

**Figure 8:**
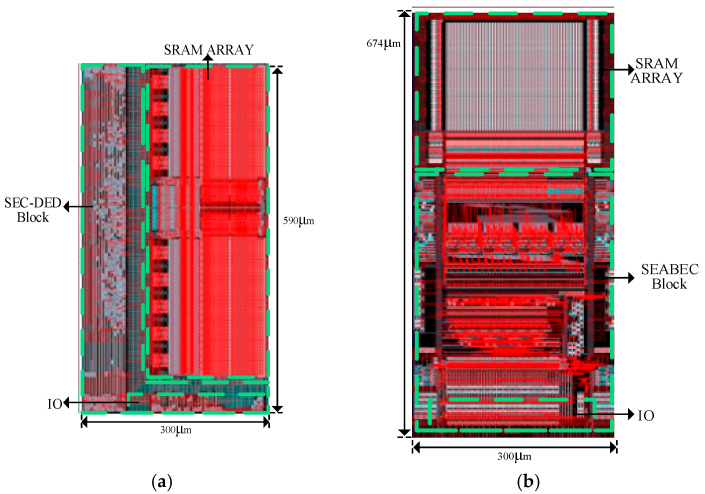
Layout of RH SRAM: (**a**) Layout of RH SRAM with SEC-DED based on static logic; (**b**) layout of RH SRAM with SEABEC based on dynamic logic.

The evaluation comprised simulations conducted with Cadence Virtuoso on a 130 nm CMOS process, operating at a 1.5 V supply voltage. As shown in Figure 8a,b, compared to the radiation-hardened SRAM with static logic-based SEC-DED, the proposed dynamic logic-based SEABEC SRAM exhibits an overall chip area overhead of 14.2%. ECC capability is strengthened through the use of SEABEC. The integrated self-refresh mechanism effectively suppresses error accumulation, supporting long-term memory reliability in radiation-prone environments.

## 4. Radiation Tests

**Figure 9:**
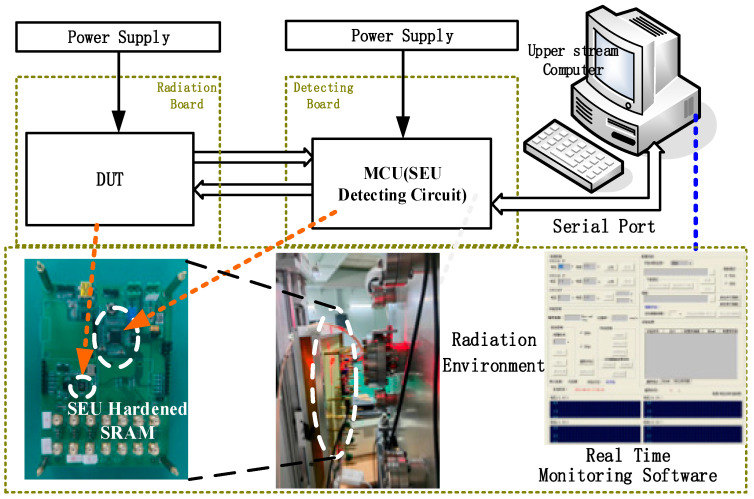
Radiation testing system.

**Figure 10:**
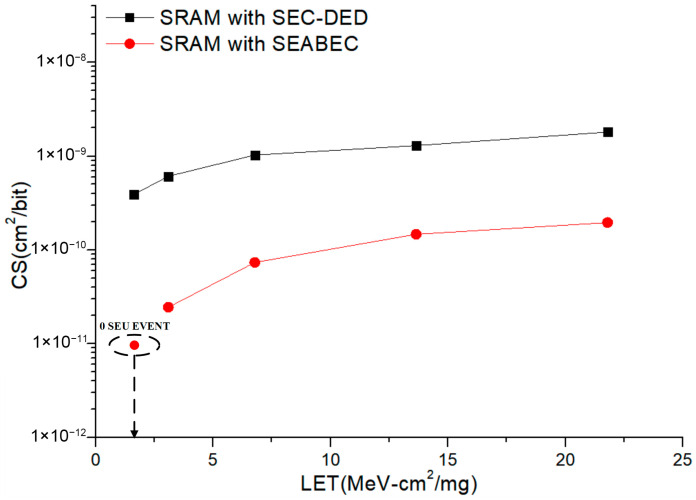
The SEU cross-section (CS) of SRAM.

Figure 9 depicts the radiation testing system and irradiation environment for the SEC-DED SRAM and SEABEC SRAM. Radiation experiments were carried out at the China Institute of Atomic Energy (CIAE). Ion irradiation parameters are summarized in Table 5 for the hardened SRAM, covering ion energy, penetration range, LET, particle flux, and total dose. Heavy-ion irradiation employed C, O, F, Si, and Ti with LET values spanning 1.63–21.8 MeV·cm^2^/mg. The experimental setup comprised test chips, SEU detection boards, and host computers for real-time monitoring and analysis of data mismatches. Radiation tests show that the SEU cross-section (CS) of the SEC-DED SRAM ranges from 2.9 × 10^−10^ cm^2^/bit to 1.8 × 10^−9^ cm^2^/bit, while the CS of SEABEC SRAM ranges from 2.4 × 10^−11^ cm^2^/bit to 1.9 × 10^−10^ cm^2^/bit, representing a reduction of one order of magnitude relative to the SEC-DED implementation, which confirms that the proposed SEABEC substantially decreases sensitivity to SEUs, as shown in Figure 10.

## 5. Conclusions

This work proposes a radiation-hardened SRAM architecture integrating a self-refresh mechanism and the SEABEC scheme, designed for high-reliability radiation-prone applications such as space electronics. By adopting extended Hamming coding and dynamic circuitry, the design achieves a 29.1% read/write speed improvement, a one-order-of-magnitude SEU cross-section reduction and nearly doubled SEU resilience compared with static logic-based RH SEC-DED SRAMs. Despite a 29.8% area overhead and 95.2% dynamic power increase for the SEABEC module, the overall chip area overhead is controlled at ~14.2%, realizing a balanced trade-off between performance, radiation tolerance, and power/area costs. Heavy-ion irradiation experiments (LET range of 1.63–21.8 MeV·cm^2^/mg) validate its superior radiation resistance, meeting the demands of high-performance microelectronic systems.

## Figures and Tables

**Table 1:** Syndrome of the new error-correction coding algorithm corresponding to single-bit error positions.

Syndrome	Single Bit Error																						No Error
	Data Error																Check Bit Error						
	a0	a1	a2	a3	a4	a5	a6	a7	a8	a9	a10	a11	a12	a13	a14	a15	p0	p1	p5	p3	p2	p4	
S0	0	1	1	1	0	1	1	1	0	0	1	0	1	1	0	1	1	0	0	0	0	0	0
S1	1	1	0	0	0	1	1	1	1	0	0	1	0	1	1	0	0	1	0	0	0	0	0
S2	0	0	1	0	1	1	0	0	0	1	0	1	1	1	0	1	0	0	0	0	1	0	0
S3	1	0	0	1	0	1	0	1	0	1	0	0	1	0	1	0	0	0	0	1	0	0	0
S4	1	0	1	0	1	0	1	0	1	0	1	0	0	0	0	0	0	0	0	0	0	1	0
S5	0	1	1	1	1	1	0	0	0	1	0	0	1	1	1	1	0	0	1	0	0	0	0

**Table 2:** Syndrome of the new error-correction coding algorithm corresponding to adjacent-bit error patterns.

Syndrome	Adjacent Bit Errors																					No Error
	a0 a1	a1 a2	a2 a3	a3 a4	a4 a5	a5 a6	a6 a7	a7 a8	a8 a9	a9 a10	a10 a11	a11 a12	a12 a13	a13 a14	a14 a15	a15 p0	p0 p1	p1 p5	p5 p3	p3 p2	p2 p4	
S0	1	0	0	1	1	0	0	1	0	1	1	1	0	1	1	0	1	0	0	0	0	0
S1	0	1	0	0	1	0	0	0	1	0	1	1	1	0	1	0	1	1	0	0	0	0
S2	0	1	1	1	0	1	0	0	1	1	1	0	0	1	1	1	0	0	0	1	1	0
S3	1	0	1	1	1	1	1	1	1	1	0	1	1	1	1	0	0	0	1	1	0	0
S4	1	1	1	1	1	1	1	1	1	1	1	0	0	0	0	0	0	0	0	0	1	0
S5	1	0	0	0	0	1	0	0	1	1	0	1	0	0	0	1	0	1	1	0	0	0

**Table 3:** Comparison of area, delay, and power consumption between static SEC-DED and dynamic SEABEC.

ECC Type	Area (µm^2^)	Delay (ns)	Power (mW)	Error Correction Capability
Static SEC-DED	30,140	2.30	2.1	Single-Error Correction and Double-Error Detection
Dynamic SEABEC	39,116	1.63	4.1	Single-Error and Adjacent -Bit Error Correction
Δ	+29.8%	−29.1%	+95.2%	The SEU resilience has been nearly doubled

**Table 4:** Comparison of existing and proposed adjacent-bit error correction codes.

Parameters	MPC	3D	AEDAC	SEC-DAEC	This Work
Data Bits, k	16	16	16	16	16
Parity Bits, r	15	15	14	8	6
Code Word, n = r + k	31	31	30	24	22
Code Rate, k/n in %	51.6	51.6	53.33	66.67	72.73
Correction Capability in Bits	2	2	2	2 (partial 3 bit adjacent errors correction)	2

**Table 5:** Characteristics of ion beams.

Ion	C	O	F	Si	Ti
Energy (MeV)	79	100	35	40	180
LET (MeV-cm^2^/mg)	1.63	3.1	6.78	13.65	21.8
Range in target (µm)	24	21	21	18.2	34.7
Flux (ions/(cm^2^ × S))	5000				
Fluence (ions)	1 × 10^7^				

## Data Availability

The data presented in this study are available on request from the corresponding author.
